# Comparative volatile profiling of *Asarum* species and MaxEnt-based habitat assessment of *Asarum sieboldii*: implications for species authentication, geographical origin traceability, and climate-informed conservation

**DOI:** 10.3389/fpls.2026.1937801

**Published:** 2026-09-01

**Authors:** Xin Jiang, Xu Qiang, Chunli Yan, Yun Jia, Xiao Zhang, Chen Chen, Guoqing Bai

**Affiliations:** 1 Xi’an Botanical Garden of Shaanxi Province (Institute of Botany of Shaanxi Province), Xi’an, Shaanxi, China; 2 Key Laboratory of Resource Biology and Biotechnology in Western China (Ministry of Education) College of Life Sciences, Northwest University, Xi’an, Shaanxi, China

**Keywords:** *A. sieboldii*, biomarkers, HS-SPME/GC-MS, species distribution, volatile metabolite

## Abstract

**Introduction:**

*Asarum sieboldii* Miq. (Aristolochiaceae) is an important medicinal plant characterized by substantial chemotypic variation. However, the potential of this variation for species authentication and geographical origin traceability, together with the effects of future climate change on its suitable habitats, remains insufficiently understood.

**Methods:**

We conducted two complementary analyses to evaluate the chemical differentiation and habitat suitability of *A. sieboldii*. Seventy two wild individuals from 12 populations, comprising seven populations of *A. sieboldii* and one population from each of five congeneric species, were profiled using HS-SPME/GC–MS and multivariate analyses. An optimized MaxEnt model was further used to identify the principal climatic factors associated with habitat suitability and to project changes in the potential suitable distribution of *A. sieboldii* under future climate scenarios.

**Results:**

A total of 131 volatile metabolites belonging to 11 chemical classes were annotated, revealing pronounced chemotypic differentiation among populations. Five candidate biomarkers, including β-bisabolene, 2-cyclohexen-1-ol acetate, 9H-fluorene derivatives, hexanal, and a putative terpenoid, consistently distinguished *A. sieboldii* from related species, whereas 12 candidate metabolites differentiated the geographical populations of *A. sieboldii* and showed potential for geographical origin traceability. The optimized MaxEnt model identified mean diurnal temperature range, annual precipitation, and mean temperature of the coldest quarter as the principal climatic predictors of habitat suitability. Under future climate scenarios, suitable habitats of *A. sieboldii* were projected to shift northward, with contraction in southern parts of the current range and limited expansion into northern regions. Shaanxi, Hubei, and Henan retained relatively stable suitable areas, whereas substantial habitat losses were projected in Jiangxi and Hunan; newly suitable areas were also projected to emerge in parts of Liaoning and Jilin Provinces.

**Discussion:**

Several candidate differential metabolites offer potential chemical markers for species authentication within *Asarum* and geographical origin traceability of *A. sieboldii*. In response to projected habitat changes, stable suitable areas should be prioritized for *in situ* conservation and long-term monitoring, newly suitable northern areas may be considered for future cultivation, and populations in contracting southern habitats should receive greater attention for *ex situ* germplasm conservation. Together, these findings provide an integrated scientific basis for species authentication, geographical origin traceability, and conservation of *A. sieboldii*.

## 1 Introduction

Traditional Chinese medicine (TCM) is widely used and relies on complex herbal materials whose multi-component chemical profiles are closely linked to therapeutic efficacy and sensory characteristics. The genus *Asarum* (Aristolochiaceae) comprises approximately 100 species, predominantly distributed across temperate regions of the Northern Hemisphere, including East Asia, North America, and Europe (Lim et al., 2018). Among these, three taxa have been widely utilized in traditional medicine: (1) *Asarum heterotropoides* Fr. Schmidt var. mandshuricum (Maxim.) Kitag. (Bei Xixin), (2) *A. sieboldii* Miq. var. seoulense Nakai (Hancheng Xixin), and (3) *A. sieboldii* Miq. (Hua Xixin, *A. sieboldii*) (Liu et al., 2021). The dried roots and rhizomes of these species constitute the primary medicinal materials collectively referred to as Xixin in Chinese pharmacopeia. Notably, the first two varieties (*A. heterotropoides* var. mandshuricum and *A. sieboldii* var. seoulense) are also regionally termed Liao Xixin in folk nomenclature (Commission, 2020). The roots contain diverse bioactive constituents, including methyleugenol, 3,4,5-trimethoxytoluene, safrole, essential oils, and ethanol extracts, which collectively contribute to their documented pharmacological effects in treating colds, headaches, toothaches, rhinorrhea, and other illnesses (Happy et al., 2025; Li et al., 2024). Although these and related taxa were extensively used to treat various ailments, their bioactive constituents inevitably varied due to interspecies differences, as well as intraspecific variations driven by climatic and other environmental factors (Lu et al., 2022). For instance, Teng et al., found the chemical compositions and contents of *Siegesbeckiae pubescens* Makino (SP), *S. orientalis L*., *S. glabrescens* Makino (SG) demonstrated significant differences (Teng et al., 2015). Such variability underscores the need for systematic characterization of medicinal components to ensure accurate species identification, reliable quality assessment, and consistent therapeutic outcomes.

Among these medicinal constituents, volatile metabolites (e.g., monoterpenes, sesquiterpenes, phenylpropanoids, and benzenoids) are of particular interest in traditional Chinese medicine, as they often contribute directly to both therapeutic efficacy and organoleptic properties. Importantly, volatile profiles can capture chemical variation at multiple biological scales—from differences among species to variation among populations within a species—thereby providing a chemical basis for both authentication and geographic traceability. At the interspecific and population levels, volatiles in *Ligusticum chuanxiong* and related species were profiled by using HS-SPME/GC-MS and reported pronounced differences both among species and among populations (Tang et al., 2022). Focusing specifically on within-species geographic differentiation, Zhang et al., identified marker metabolites that distinguished geographic chemotypes of *Atractylodes lancea* rhizomes across China, demonstrating the utility of volatile signatures for origin authentication (Zhang et al., 2023). Volatile-derived markers can also resolve closely related taxa, as shown by Ji et al., who identified 7-hydroxycoumarin and Z-ligustilide as elevated in *Angelica sinensis* compared to other *Angelica* species, providing a basis for species authentication (Ji et al., 2025). Despite these advances, comparable systematic volatile-metabolite profiling in *Asarum*—including robust markers for both species discrimination and within-species origin traceability—remains scarce, limiting progress in authentication, quality control, and geographic traceability.

Accurately determining the geographic origins of TCMs is critically important because medicinal materials from core production areas are generally perceived to exhibit superior quality compared to those from peripheral regions and are therefore more susceptible to substitution and adulteration. In practice, geographic traceability is increasingly addressed through metabolomics-based strategies that couple chemical profiling with multivariate statistics or feature-selection algorithms to discover origin-discriminatory markers. Depending on the chemical space of interest, complementary analytical platforms are commonly applied: HPLC/LC–MS is widely used to characterize non-volatile or thermolabile constituents, whereas GC–MS—particularly HS-SPME/GC–MS—is well suited for capturing volatile signatures that directly shape organoleptic properties and can provide sensitive markers for authentication. For instance, Yang et al. used HS-SPME/GC–MS to comparatively characterize the volatile profiles of the medicinal herb *Prunella vulgaris* collected from five geographical regions in China, revealing clear origin-related differences in volatile composition (Yang et al., 2013). Similarly, origin-dependent metabolic signatures have been reported for *Lycii Fructus* (goji berry), a medicinal and edible plant product, further demonstrating the utility of metabolite profiles for geographical origin discrimination (Han et al., 2025). Although many volatile compounds in various medicinal plants have been extensively characterized, comprehensive profiling in *Asarum* species, especially for identifying reliable species- and region-specific markers, is still limited.

In addition to quality assurance, understanding how medicinal plant resources respond to environmental drivers and future climate change is essential for sustainability. Over the past decades, anthropogenic greenhouse gas emissions, particularly carbon dioxide (CO_2_), have driven striking global climate change, predominantly characterized by rising temperatures (Montzka et al., 2011). This exerts profound pressure on biodiversity, particularly affecting species with narrow habitats, and often leads to habitat fragmentation, population decline, and elevated extinction risk. Consequently, predicting species’ distributional shifts under climate change is essential, as it elucidates potential adaptation mechanisms and informs targeted conservation and sustainable-use strategies. To quantify such distributional changes, Species Distribution Models (SDMs) – such as the Bioclimatic Analysis System (BIOCLIM), the Genetic Algorithm for Rule-set Production (GARP), and the Maximum Entropy (MaxEnt) model – have been extensively employed as key analytical tools (Wang et al., 2023). Among these, MaxEnt has proven particularly reliable for modeling species with limited occurrence records, owing to its robust performance with small sample sizes and its ability to identify key bioclimatic constraints (Sang et al., 2022). For instance, Lv et al. applied MaxEnt to predict the potential distribution of four endangered Saxifragaceae species, projecting a likely southward shift in their ranges in response to ongoing climate change (Lv et al., 2025). Similarly, Hu et al. used MaxEnt to model the future distribution of 17 endangered orchid species on the Qinghai-Tibetan Plateau under two climate scenarios for the 2050s and 2070s and their findings indicated that species richness was highest in the southeastern part of the plateau and declined with increasing elevation across scenarios, underscoring the southeastern region as a priority for conservation (Hu et al., 2022). By applying the MaxEnt model, researchers can identify potential shifts in suitable habitats under climate change and evaluate regions at risk of habitat contraction, thereby providing a scientific basis for the sustainable use and management of medicinal plant resources.

In this study, we integrated headspace solid-phase microextraction coupled with gas chromatography-mass spectrometry (HS-SPME/GC-MS) with multivariate statistical analysis to characterize the volatile metabolites of *Asarum* species. A total of 72 samples representing *A. sieboldii* and five closely related species were collected from geographically distinct populations across eight provinces in China. The objectives were to identify key discriminatory metabolites for (1) species authentication among related *Asarum* taxa and (2) geographical origin traceability of *A. sieboldii*. In addition, species distribution modeling was used to evaluate potential changes in suitable habitats under future climate scenarios, thereby providing a basis for climate-informed conservation strategies for this medicinal plant. Collectively, our findings provide a volatile-metabolite basis for species authentication and geographical origin traceability, while offering ecological evidence to support its conservation and cultivation planning under future climate change.

## 2 Materials and methods

### 2.1 Sample and occurrence collection

A total of 72 wild *Asarum* individuals were collected from 12 populations across seven Chinese provinces—Liaoning, Henan, Hubei, Shandong, Shaanxi, Gansu, and Guangxi—between June and August 2018. Of the 12 populations, seven populations of *A. sieboldii* Miq. were distributed across five provinces (Liaoning, Henan, Hubei, Shandong, and Shaanxi Provinces), whereas each of the remaining five populations represented one congeneric species: *A. himalaicum*, *A. renicordatum*, *A. splendens*, *A. macranthum*, or *A. geophilum*. Thus, the study included six *Asarum* species in total, with *A. sieboldii* represented by multiple geographical populations and each congeneric species represented by a single population. Population codes were assigned according to the corresponding collection localities, such as BX for Benxi, Liaoning Province, and LCH for Luanchuan, Henan Province (**Table 1**). None of the plant species included in this study were listed as protected species under the applicable national or local conservation regulations at the time of collection. Accordingly, sample collection and use complied with the relevant legal and institutional requirements. All samples were planted at Xi’an Botanical Garden of Shaanxi Province (Shaanxi, China, 34°120 N, 109°10 E) under shared cultivation conditions for further studies and species identification was confirmed by Dr. Bai. Voucher specimens for the *Asarum* species were deposited in Xi’an Botanical Garden of Shaanxi Province (Xi’an, China) with accession number XABS20250337 (*Asarum sieboldii*), XABS20250338 (*Asarum himalaicum*), XABS20250339 (*Asarum renicordatum*), XABS20250340 (*Asarum splendens*), XABS20250341 (*Asarum macranthum*), XABS20250342 (*Asarum geophilum*). Fresh root tissues were collected, immediately frozen in liquid nitrogen for subsequent metabolite analysis.

**Table 1:** Information of *Asarum* species rhizome samples.

Species	Location	Population code	Longitude	Latitude
*A. sieboldii*	Benxi, Liaoning	BX	123.73	41.32
*A. sieboldii*	Luanchuan, Henan	LCh	111.69	34.00
*A. sieboldii*	Enshi, Hubei	EnH	109.49	30.35
*A. sieboldii*	Laoshan, Shandong	LSh	120.60	36.22
*A. sieboldii*	Ankang, Shaanxi	HB	108.78	33.85
*A. sieboldii*	Ningqiang, Shaanxi	NQ	107.7	34.05
*A. sieboldii*	Huayin, Shaanxi	HY	110.05	34.50
*A. himalaicum*	Lixian, Gansu	LX	105.07	34.20
*A. renicordatum*	Enshi, Hubei	EnM	109.08	30.40
*A. splendens*	Enshi, Hubei	EnQ	109.16	29.87
*A. macranthum*	Enshi, Hubei	EnD	109.87	30.37
*A. geophilum*	Hechi, Guangxi	HCh	108.08	24.69

To assess the potential distribution of *A. sieboldii* under future climate change, occurrence records were compiled from multiple sources: the Chinese Virtual Herbarium (https://www.cvh.ac.cn/, accessed on 5 September 2025), the National Specimen Information Infrastructure (https://nsii.org.cn/2017/home.php, accessed on 5 September 2025) the Global Biodiversity Information Facility (https://www.gbif.org/, accessed on 5 September 2025), and field surveys conducted by our team. A total of 279 occurrence points were initially obtained. To minimize spatial sampling bias and reduce redundancy, we used ENMtools to filter the records, retaining only one occurrence point per 5 km × 5 km grid cell (Warren et al., 2021). This resulted in 115 spatially independent points for subsequent modeling (**Supplementary Figure 1**, **Supplementary Table 1**). Environmental variables included 19 bioclimatic parameters obtained from WorldClim (https://worldclim.org/, accessed on 15 September 2024) at ~5 km resolution, along with 5 soil variables derived from the Harmonized World Soil Database (HWSD; https://gaez.fao.org/pages/hwsd, accessed on 2 December 2024). Slope and aspect were generated from a digital elevation model using ArcGIS v10.8. All variables were resampled to a consistent spatial resolution of 2.5 minutes (**Supplementary Table 2**). For future projections, we employed the BCC-CSM2-MR global climate model, which has been shown to perform well for climatic simulations in China (Debella-Gilo and Etzelmüller, 2009). Two contrasting shared socioeconomic pathways (SSPs) were selected to represent different greenhouse gas emission scenarios: SSP1-2.6 (low emissions) and SSP5-8.5 (high emissions). These were used to model the potential distribution of *A. sieboldii* future climate conditions.

### 2.2 Variables screen and niche modeling analysis

To minimize multicollinearity among environmental variables and improve model reliability, variable selection was conducted based on two criteria: the percent contribution of each variable derived from preliminary MaxEnt runs, and pairwise Pearson correlation coefficients calculated using IBM SPSS Statistics V26.0. When two variables exhibited a correlation coefficient exceeding |0.8|, only the variable with the higher contribution was retained for subsequent analysis. After this screening process, 11 variables were selected for final modeling. All environmental layers were visualized by using TBtools (Chen et al., 2023) (**Supplementary Figure 2**). MaxEnt model complexity was optimized using the ENMeval package in R (Kass et al., 2021). Six feature-class combinations (L, LQ, H, LQH, LQHP, and LQHPT) and eight regularization multiplier (RM) values ranging from 0.5 to 4.0 at intervals of 0.5 were evaluated, resulting in 48 candidate models (**Supplementary Table 3**) (Yang et al., 2024). A total of 10,000 background points were randomly sampled from environmentally valid raster cells, and spatial block cross-validation was used to evaluate candidate models. Model selection was primarily based on the corrected Akaike information criterion (AICc) and ΔAICc. Models with ΔAICc ≤ 2 were considered well supported, and the model with the lowest AICc value (ΔAICc = 0) was selected as the optimal model (Liu et al., 2024b). The optimal parameter combination was linear, quadratic, hinge, product, and threshold features (FC = LQHPT) with a regularization multiplier of 3.5 (RM = 3.5).

The optimized parameter combination was subsequently implemented in MaxEnt version 3.4.1 to predict the current and future habitat suitability of *A. sieboldii*. The subsample method was used with 10 replicate runs. In each replicate, 75% of the occurrence records were randomly assigned to model training and the remaining 25% were used for independent testing (Liu et al., 2022). Model predictive performance was evaluated using the area under the receiver operating characteristic curve (AUC), and the relative contribution and permutation importance of environmental variables were further assessed using the jackknife test. Model outputs were imported into ArcGIS v10.8 for spatial analysis. Potential habitat suitability was reclassified into four categories—unsuitable, low suitability, moderate suitability, and high suitability—using the natural breaks (Jenks) classification method (Jenks, 1967). SDMtoolbox was employed to calculate the dynamic change, the suitable habitats in the future compared with the current (Brown, 2014). The area of each suitability class was calculated for the current and future periods to compare changes over time. Additionally, to examine spatial shifts in suitable habitat, future projections were overlapped with the current potential distribution to identify regions of habitat expansion, contraction, and stability.

### 2.3 Isolation and analysis of the volatiles by HS-SPME and GC-MS

Volatile compounds in the stem tissues of the Asarum samples were analyzed by headspace solid-phase microextraction coupled with gas chromatography–mass spectrometry (HS-SPME–GC–MS) using an Agilent 7890B–7000D GC–MS system (Agilent Technologies, Palo Alto, CA, USA). Fresh stem tissues were immediately frozen in liquid nitrogen, stored at −80 °C, and ground into a fine powder under liquid nitrogen before analysis. For each extraction, approximately 1.0 g of accurately weighed powdered sample was transferred into a 20 mL headspace vial containing saturated NaCl solution and 10 μL of internal-standard solution. The vial was sealed with a crimp-top cap fitted with a TFE–silicone headspace septum. A 65 μm divinylbenzene/carboxen/polydimethylsiloxane fiber (DVB/CAR/PDMS; Supelco, Bellefonte, PA, USA) was conditioned at 250 °C for 5 min before extraction. Each sample was equilibrated at 60 °C for 10 min, after which the fiber was exposed to the vial headspace at 60 °C for 20 min. After extraction, the fiber was inserted into the GC injection port and desorbed at 250 °C for 5 min in splitless mode, followed by post-injection fiber conditioning for 5 min. Chromatographic separation was performed on a DB-5MS capillary column (30 m × 0.25 mm × 1.0 μm; 5% phenyl-polymethylsiloxane), with helium as the carrier gas at a constant flow rate of 1.0 mL/min. The injector temperature was maintained at 250 °C. The oven temperature was initially maintained at 40 °C for 5 min, increased to 280 °C at 6 °C/min, and held at 280 °C for 5 min. Mass spectrometric detection was performed in electron-ionization mode at 70 eV. The quadrupole, ion-source, and transfer-line temperatures were maintained at 150, 230, and 280 °C, respectively.

Mass spectra were acquired over the range of m/z 30–350 at 1 s intervals. A pooled quality-control sample was prepared by combining portions of the study samples and was analyzed after every ten study samples to monitor analytical reproducibility. Analytical stability was evaluated by comparing the retention times and signal intensities in the overlaid total-ion chromatograms of the QC samples. Volatile compounds were putatively annotated by comparing the acquired electron-ionization mass spectra with entries in the MetWare Gas Chromatography database (MWGC 1.0; Wuhan Metware Biotechnology Co., Ltd., Wuhan, China), which incorporates reference information from the NIST and Wiley mass-spectral libraries. Only compounds with a mass spectral match factor of at least 70 were retained. Experimental linear retention indices (RI*) were determined using a homologous series of n-alkanes analyzed under the same chromatographic conditions, as previously described (Li et al., 2025). Compound annotation was based on both the mass spectral matching results and the agreement between the experimental RI* values (Yang et al., 2026).

### 2.4 Data processing and statistical analysis

Raw GC–MS data were processed using MassHunter software (Agilent Technologies) for peak extraction, integration, and correction. Information on the mass-to-charge ratio, retention time, and peak area of each detected feature was obtained. Features were retained when the proportion of missing values did not exceed 50% within at least one experimental group or across all samples. The retained peak areas were normalized against the internal standard, and the resulting normalized metabolite-abundance matrix was used for subsequent statistical analyses. All identified metabolites were annotated using Kyoto Encyclopedia of Genes and Genomes (KEGG) pathway databases (Altermann and Klaenhammer, 2005). Differential metabolites were visualized and analyzed using multiple statistical approaches. Heatmaps were generated using the ggplot2 package in R to display expression differences of metabolites among groups. To reduce data dimensionality and elucidate complex inter-sample relationships, an unsupervised multivariate analysis, principal component analysis (PCA), was employed. The resulting principal component scores facilitated the evaluation of variation between groups (Liu et al., 2024c). For superior group separation, a supervised multivariate statistical approach was adopted: partial least squares-discriminant analysis (PLS-DA) alongside orthogonal PLS-DA (OPLS-DA). The modeling was executed utilizing the SIMCA 14.1 software suite. This involved constructing a robust model that maps the relationship between the observed metabolite abundance and the known group labels, ultimately yielding a powerful predictor for sample grouping (Abdelhafez et al., 2020). Model performance was assessed by R²X and R²Y, representing the explained variance of the X and Y matrices, respectively, while Q² values closer to 1 indicated superior predictive capability. The robustness of the species-level multiclass PLS-DA model and each of the five species-level pairwise OPLS-DA models was evaluated using 200 permutation tests. In each permutation, the class labels were randomly reassigned while the metabolite-abundance matrix remained unchanged. The R²Y and Q² values of the original models were compared with those obtained from models generated using randomly permuted class labels. The corresponding pR²Y and pQ² values were calculated, and values below 0.05 were considered to indicate that the original model performed significantly better than the permuted models. Variables with VIP (variable importance) > 1 were considered important contributory to group discrimination (Liu et al., 2024c).

### 2.5 Screening and analysis of candidate differential metabolites

Candidate differential metabolites were screened in both interspecific comparisons between *A. sieboldii* and each of the five related species and intraspecific comparisons among geographically distinct populations of *A. sieboldii*. Metabolites were considered candidates when they simultaneously met the criteria of |log_2_FC| > 0.584, VIP > 1, and *P* < 0.05. VIP values were derived from the corresponding supervised multivariate models and were used to assess the contribution of individual metabolites to group discrimination. Statistical significance for each pairwise comparison was assessed using Student’s t-test, with *P* < 0.05 used as the threshold (Feng et al., 2025). Volcano plots were generated for each pairwise comparison to visualize the magnitude of metabolite changes and their statistical significance. UpSet plots were further used to summarize shared and unique candidate differential metabolites across comparisons and were generated using the UpSetR package (Conway et al., 2017) in R.

## 3 Results and discussion

### 3.1 Metabolomic profiling of *Asarum* volatiles across geographic gradients

**Figure 1:**
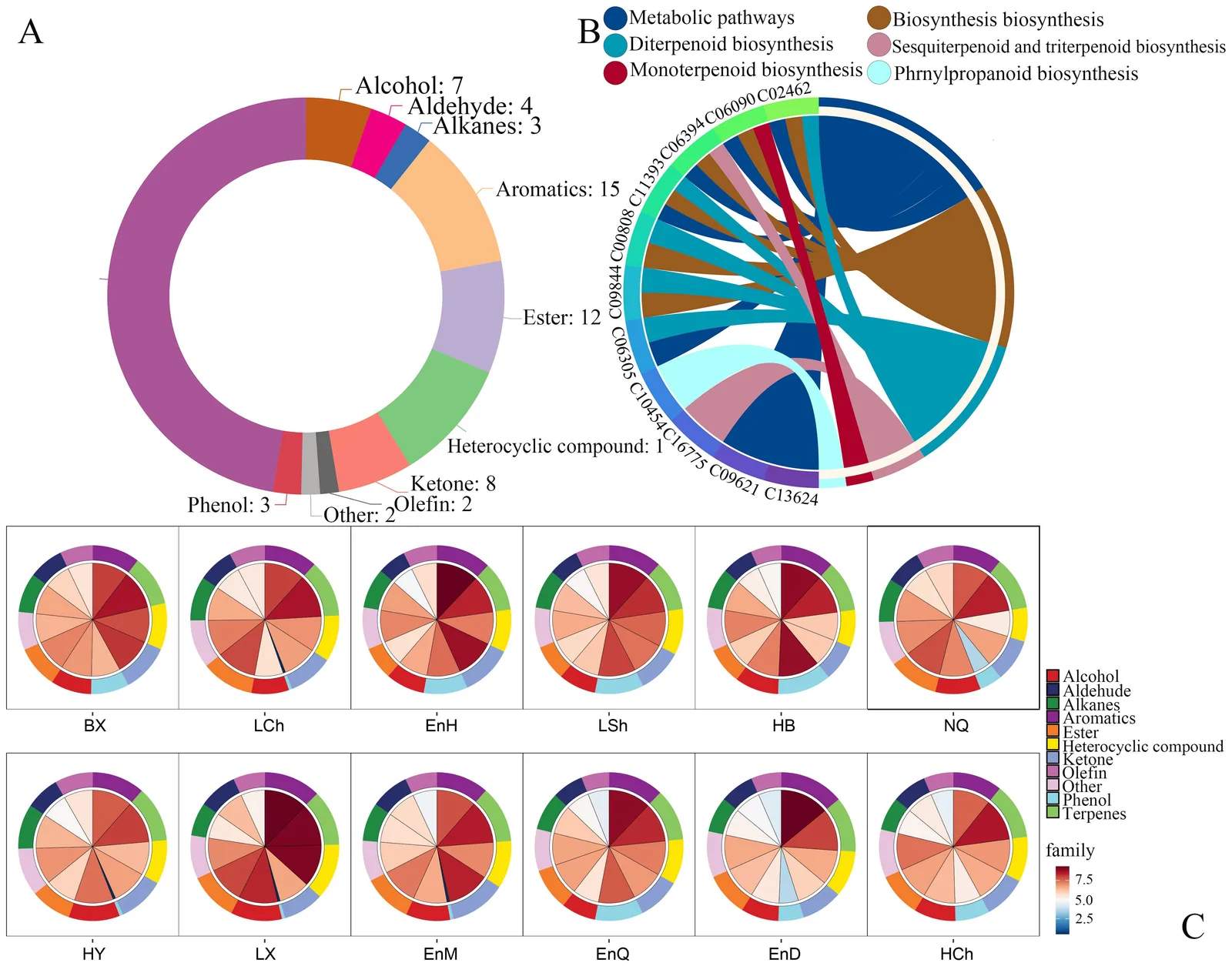
Metabolic profiling of QC samples and natural populations. **(A)** Relative abundance distribution of all detected metabolites in quality control (QC) samples. **(B)** KEGG enrichment of volatile metabolites. **(C)** Pie charts showing metabolite composition proportions across 12 sampled populations.

To comprehensively analyze the aroma-related metabolites of all samples, we employed headspace solid-phase microextraction coupled with gas chromatography–mass spectrometry (HS-SPME-GC-MS), a widely used approach in metabolomic studies. This technique enabled the detection and characterization of a broad spectrum of volatile substances, thereby facilitating comparisons of their compositional differences among the experimental groups. Analysis revealed 131 volatile metabolites, which were further categorized into 11 groups according to their chemical structures: terpenes (62 compounds, 47.3%), alcohols (7), aldehydes (4), alkanes (3), aromatics (15), esters (12), heterocyclic compounds (13), ketones (8), olefins (2), phenols (3), and others (2) (**Figure 1A**, **Supplementary Table 4**). Terpenoids represented the largest group of volatile metabolites in this study. Similar patterns were described by Guan et al., who observed that terpenoids together with heterocyclic compounds accounted for the major flavor components in cherry tomatoes of different colors (Guan et al., 2025). The predominance of terpenoids can be attributed to their diverse ecological functions, particularly their well-documented roles in plant defense mechanisms (Holopainen et al., 2025). These compounds are known to mediate responses to both abiotic stress (e.g., temperature fluctuations, drought) and biotic stress (e.g., herbivory, pathogen attack), enhancing plant fitness under varying environmental conditions (Boncan et al., 2020). All identified volatile metabolites were annotated by using Kyoto Encyclopedia of Genes and Genomes (KEGG) pathway databases, and were mainly enriched in metabolic pathways, biosynthesis of secondary metabolites, monoterpenoid biosynthesis, sesquiterpenoid and triterpenoid biosynthesis, diterpenoid biosynthesis, phenylpropanoid biosynthesis (**Figure 1B**). Comparative analysis of volatile composition across different groups revealed distinct metabolic profiles (**Figure 1C**). While the majority of the 11 volatile compound categories remained relatively stable across all groups, their relative abundances still exhibited subtle variations. Notably, the phenol content was significantly lower in the Lch, HY, LX, and EnM groups compared to other groups. This divergence suggests group-specific metabolic adaptations, likely influenced by environmental factors, given that phenolic compounds are well-established as key stress-responsive metabolites in plants (Saini et al., 2024). Phenolics play a crucial role in mitigating abiotic and biotic stresses, contributing to both defense mechanisms (Ultraviolet (UV) protection, pathogenic infections, defense against herbivores and pathogens) (Ahlawat et al., 2024).

### 3.2 Multivariate patterns of metabolomic diversity in *Asarum* populations

**Figure 2:**
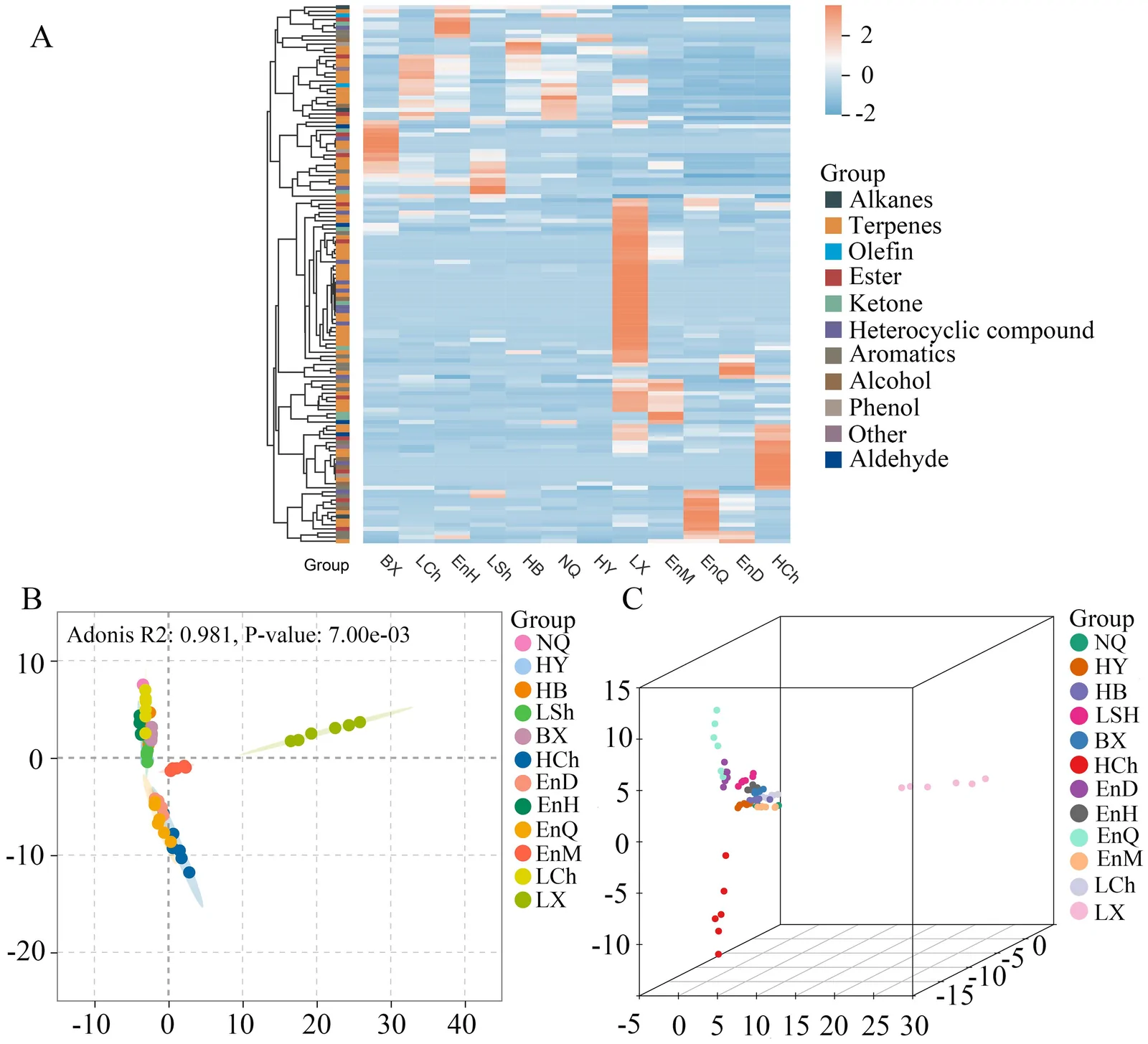
Multivariate analysis of metabolomic variation across *Asarum* populations. **(A)** Heatmap with hierarchical clustering of volatile components from different populations; **(B)** 2D PCA plots of 12 populations; **(C)** 3D PCA plots of all populations.

Comprehensive multivariate statistical analyses were used to investigate the difference of volatile metabolites among the 12 groups. Hierarchical clustering analysis of the heatmap revealed a distinct bipartite grouping pattern: samples from BX to HY clustered together, while those from LX to HCh formed a separate cluster (**Figure 2A**). Notably, this classification aligned precisely with the taxonomic distinction between *A. sieboldii* and other related species. The consistency between chemical profiling and species classification further supports the reliability of our findings. Interestingly, LX (*A. himalaicum*) exhibited the most distinct metabolic profile and its metabolic signature differed not only from its closely related congeners (HCh: *A. renicordatum*, EnQ: *A. splendens*, EnD: *A. macranthum*, HCh: *A. geophilum*) within the same cluster but also from the opposing cluster (BX_HY, *A. sieboldii*). This unique metabolic divergence may reflect specialized biosynthetic pathways in LX, potentially driven by ecological adaptations (e.g., distinct pollination strategies or microenvironmental pressures) or evolutionary divergence in secondary metabolism.

Principal component analysis (PCA) corroborated these findings, revealing a consistent bipartite clustering pattern (LX/HCh vs. BX/HY) across both 2D and 3D projections. Notably, the LX group exhibited metabolic distinctness from both clusters (**Figure 2B**), attributable to its significantly higher terpenoid content (**Figure 2A**), which drove its divergence along primary principal components. The 3D PCA (PC1: 33.87%, PC2: 15.28%, PC3: 10.2%; cumulative variance: 59.35%) further resolved the metabolic relationships: while the bipartite clustering persisted, both LX and HCh occupied unique subspaces (**Figure 2C**). This spatial separation reflects their divergent chemical compositions—LX’s terpenoid enrichment and HCh’s distinct metabolite profile collectively underpin their non-overlapping positions in the PCA space.

### 3.3 Volatile metabolite profiling and identification of differential markers for species differentiation

**Figure 3:**
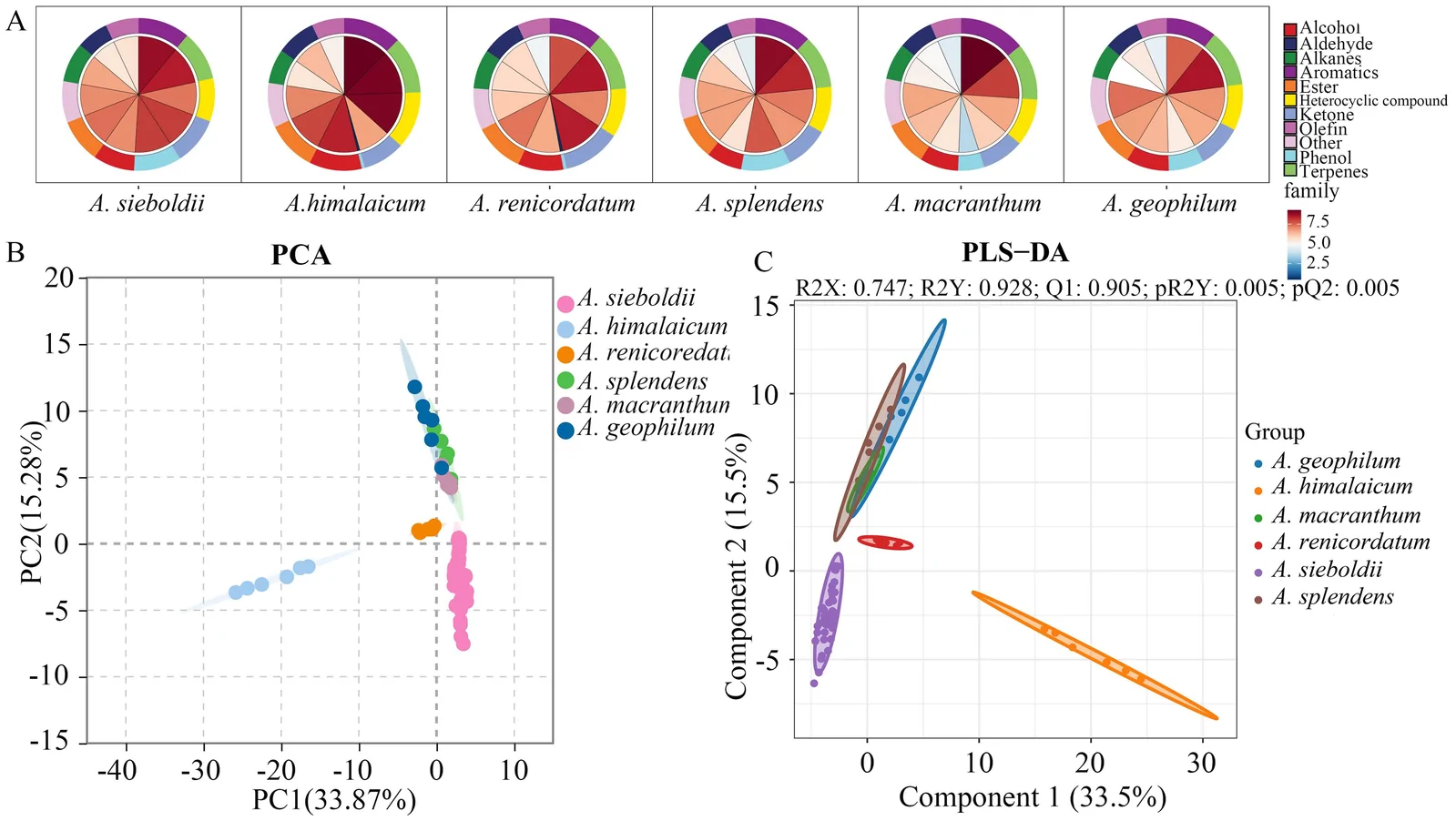
Multivariate analysis of metabolomic variation across *Asarum* species. **(A)** Abundance distribution in different species; **(B)** 2D PCA plots of six species; **(C)** PLS_DA analysis.

The volatile metabolite profiles across all studied species (*A. himalaicum*, *A. renicordatum*, *A. geophilum*, *A. splendens*, *A. macranthum*, and *A. sieboldii*) were systematically characterized. Among the 11 identified metabolite categories, phenolic compounds were significantly reduced in *A. himalaicum* and *A. renicordatum* compared to other species (**Figure 3A**). PCA revealed distinct clustering patterns, with *A. geophilum*, *A. splendens*, and *A. macranthum* forming a cohesive group, while *A. himalaicum* exhibited clear metabolic divergence (**Figure 3B**). To identify species-specific metabolic markers, partial least squares-discriminant analysis (PLS-DA) was performed. The model demonstrated strong discriminatory power (R²Y = 0.928, Q² = 0.905), confirming robust separation among all species with superior fitting and predictive abilities (**Figure 3C**). A VIP ≥ 1.0 and *p* < 0.05 were defined as the two conditions for discriminating VOCs for six species (Liang et al., 2024). Finally, metabolomic analysis identified 30 discriminatory metabolites with VIP scores exceeding 1.0 (**Supplementary Figure 3**). Among these, four compounds demonstrated particularly strong discriminatory power with VIP values over 1.75. Among these, benzene derivatives, levomenol, ethanone, and isoelemicin showed the highest VIP values and contributed most strongly to species discrimination. These key metabolites, along with the remaining 26 markers, collectively account for the observed interspecific metabolic differentiation (**Supplementary Figure 3**). To further resolve candidate discriminatory metabolic signatures among species, pairwise OPLS-DA analyses were performed. All models showed excellent discrimination (*A. geophilum* vs. *A. sieboldii*: R²Y > 0.995, Q² > 0.994; *A. himalaicum* vs. *A. sieboldii*: R²Y > 0.996, Q² > 0.9948; *A. macranthum* vs. *A. sieboldii*: R²Y > 0.9961, Q² > 0.9946; *A. renicordatum* vs. *A. sieboldii*: R²Y > 0.9975, Q² > 0.9956; and *A. splendens* vs. *A. sieboldii*: R²Y > 0.9951, Q² > 0.9944), with clear separation in the score plots (**Supplementary Figure 4**). To further evaluate model robustness and potential overfitting, 200 permutation tests were performed for each pairwise OPLS-DA model. In all comparisons, the R²Y and Q² values of the original models were substantially higher than those obtained from models with randomly permuted class labels, with pR²Y = 0.005 and pQ² = 0.005, providing no apparent evidence of overfitting based on the permutation tests (**Supplementary Figure 5**, **Supplementary Table 5**).

**Figure 4:**
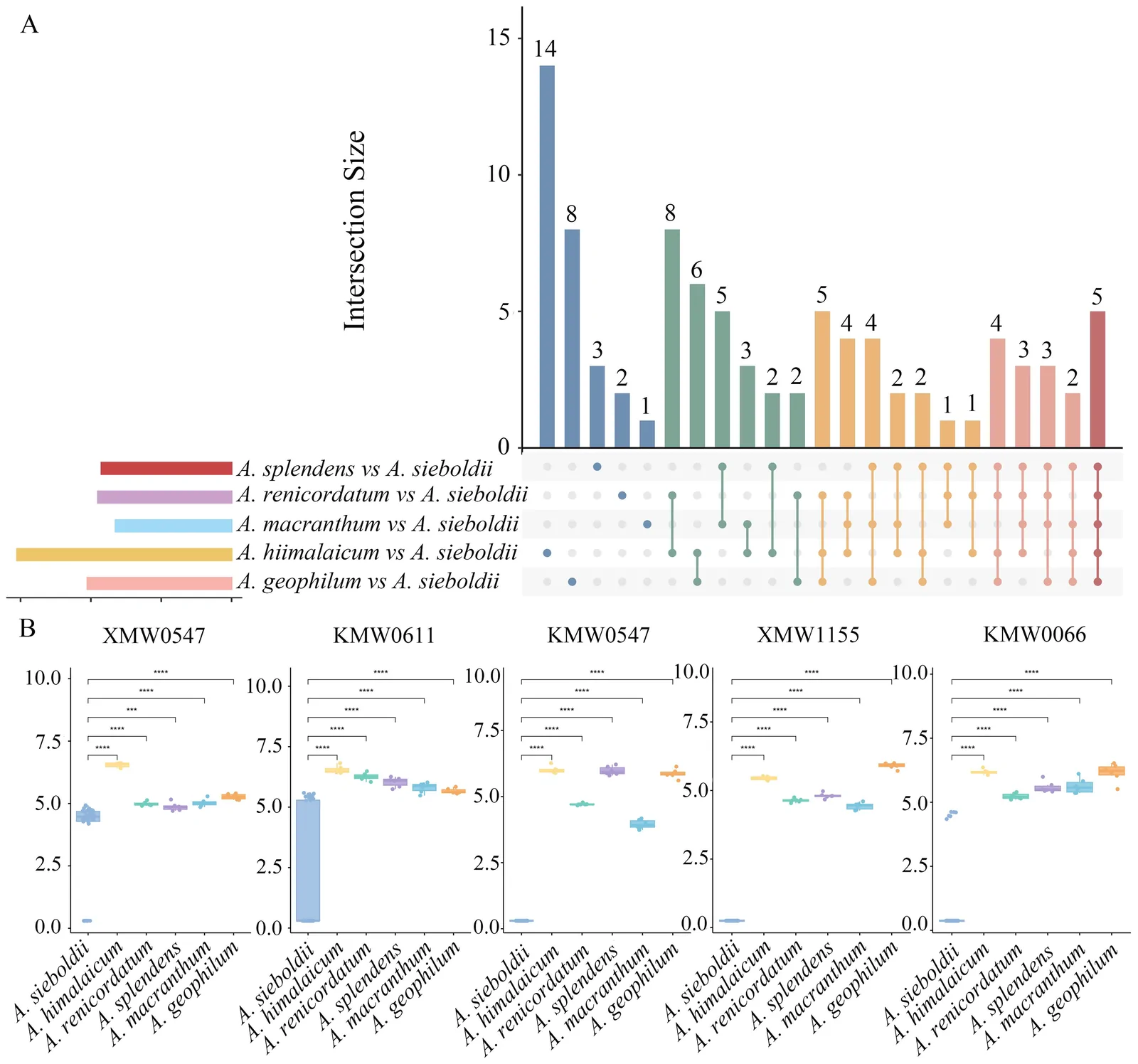
Comparative analysis of differential metabolites using an UpSet plot and boxplots. **(A)** Intersection of differential metabolites across species. **(B)** Statistical validation of differential metabolites.

Systematic comparative analysis revealed significant metabolic divergence between *A. sieboldii* and five related species (*A. geophilum*, *A. himalaicum*, *A. macranthum*, *A. renicordatum*, and *A. splendens*), as demonstrated by pairwise volcano plot analysis (**Supplementary Figure 6**). The comparison between *A. geophilum* and *A. sieboldii* identified 40 differential metabolites (DMs), of which 35 (87.5%) were up-regulated in *A. sieboldii*, while only 5 (12.5%) were down-regulated (**Supplementary Figure 6**, **Supplementary Table 6**). Similarly, *A. macranthum* displayed 32 DMs compared to *A. sieboldii*, consisting of 25 (78.1%) up-regulated and 7 (21.9%) down-regulated metabolites (**Supplementary Figure 6**, **Supplementary Table 7**). The *A. renicordatum* versus *A. sieboldii* comparison yielded 37 DMs, including 35 (94.6%) up-regulated compounds (**Supplementary Figure 6**, **Supplementary Table 8**), while *A. splendens* showed 36 DMs, with 31 (86.1%) up-regulated (**Supplementary Figure 6**, **Supplementary Table 9**). Strikingly, *A. himalaicum* exhibited complete unidirectional metabolic differentiation relative to *A. sieboldii*, with all 61 detected DMs showing up-regulation (**Supplementary Figure 6**, **Supplementary Table 10**). Collectively, these results highlight *A. sieboldii*’s distinct metabolic profile, characterized by predominant up-regulation of specialized metabolites, with the most pronounced divergence observed in its comparison with *A. himalaicum*. The Dem_Upset plot analysis revealed five candidate volatile metabolites across all pairwise comparisons with (−)-1, 2, 2α, 3, 3, 4, 6, 7, 8, 8α-decahydro-2α, 7, 8-trimethylacenaphthylene, β-bisabolene, 2-cyclohexen-1-ol, 2-methyl-5-(1-methylethenyl)-, acetate, 1,9-dimethyl-9H-fluorene, and hexanal (**Figure 4A**, **Table 2**).

**Table 2:** Five shared metabolites in *A. sieboldii* and five related species.

Index	Compounds	Exact mass (Da)	Formula	Class	CAS	RT	RI
XMW0547	(-)-1,2,2.alpha.,3,3,4,6,7,8,8.alpha.-decahydro-2.alpha.,7,8-trimethylacenaphthylene	204.188	C15H24	Terpenes	1000365-86-7	21.86	1368.06
KMW0611	.beta.-Bisabolene	204.188	C15H24	Terpenes	495-61-4	25.82	1524.26
KMW0547	2-Cyclohexen-1-ol, 2-methyl-5-(1-methylethenyl)-, acetate	194.131	C12H18O2	Ester	97-42-7	20.75	1326.61
XMW1155	9H-Fluorene, 1,9-dimethyl-	194.11	C15H14	Aromatics	23.16	1417.47	77.56
KMW0066	Hexanal	100.089	C6H12O	Aldehyde	66-25-1	5.5	793.19

Hexanal, a highly volatile C6 aldehyde, is a well-characterized plant volatile organic compound (VOC) produced through the lipoxygenase pathway in response to tissue damage (Espino-Díaz et al., 2016). β-Bisabolene is a sesquiterpene known for its distinctive balsamic scent. It holds regulatory approval in Europe for use as a flavoring agent in food products (Nuutinen, 2018). Furthermore, its antioxidant efficacy has been observed in various spice-derived essential oils, including those extracted from black pepper (*Piper nigrum*), Javanese long pepper (*Piper retrofractum*), and red ginger (*Alpinia purpurata*) (Pramitha et al., 2025). The acetate derivative 2-cyclohexen-1-ol, 2-methyl-5-(1-methylethenyl)-, acetate has been reported to exhibit inhibitory activity in essential oils, particularly in sweet orange peel (Bukari et al., 2025). Similar antimicrobial properties have been observed for structurally related compounds, including limonene in citrus essential oils (Patkowska, 2006). The widespread use of traditional Chinese medicines (TCMs) has garnered significant scientific interest, particularly regarding their pharmacological properties and quality control (Ma et al., 2022a). Interspecies identification represents a fundamental requirement for ensuring the authenticity and therapeutic efficacy of medicinal materials, as even phylogenetically related species may possess markedly distinct bioactive compound profiles. A notable example can be found in the *Erigeron* genus: *Erigeron breviscapus* (EB) and *Erigeron multiradiatus* (EM) are both referred to as “meiduoluomi” in traditional Tibetan medicine and have been historically used to treat plague and epidemic diseases. However, in traditional Chinese medicine, only EB is clinically applied for cerebrovascular disorders, post-stroke hemiplegia, coronary artery disease, chest congestion, and angina pectoris. This divergence in therapeutic applications between the two species across different medical systems highlights the importance of accurate species identification (Ma et al., 2022a). Furthermore, comparative metabolomic analysis revealed that the content of five key candidate metabolites in *A. sieboldii* was significantly lower than in the other five investigated species (**Figure 4B**). This unique chemical composition indicates that the identified metabolites could function as candidate indicators for distinguishing species, thereby offering a theoretical framework for quality control in herbal medicine.

### 3.4 Intra-specific metabolic variation in Huaxixin populations

To determine the compositional differences in volatile metabolites among different *A. sieboldii* sample groups, gas chromatography-mass spectrometry (GC-MS) analysis was performed. The results indicated that the levels of phenol were notably lower in the LCh and HY groups relative to all other groups examined (BX, EnH, LSh, HB, NQ). Notably, the content in LX (*A. himalaicum*) and EnM (*A. renicordatum*) were significantly lower as well, indicating the varied phenol contents more likely due to the effect of environments rather than species-specific (**Figure 1C**). Furthermore, the widespread detection of alcohols, aldehydes, and esters in all investigated areas underscores their ubiquitous role in shaping metabolic profiles, consistent with earlier results described by Xiao et al (Xiao et al., 2017).

**Figure 5:**
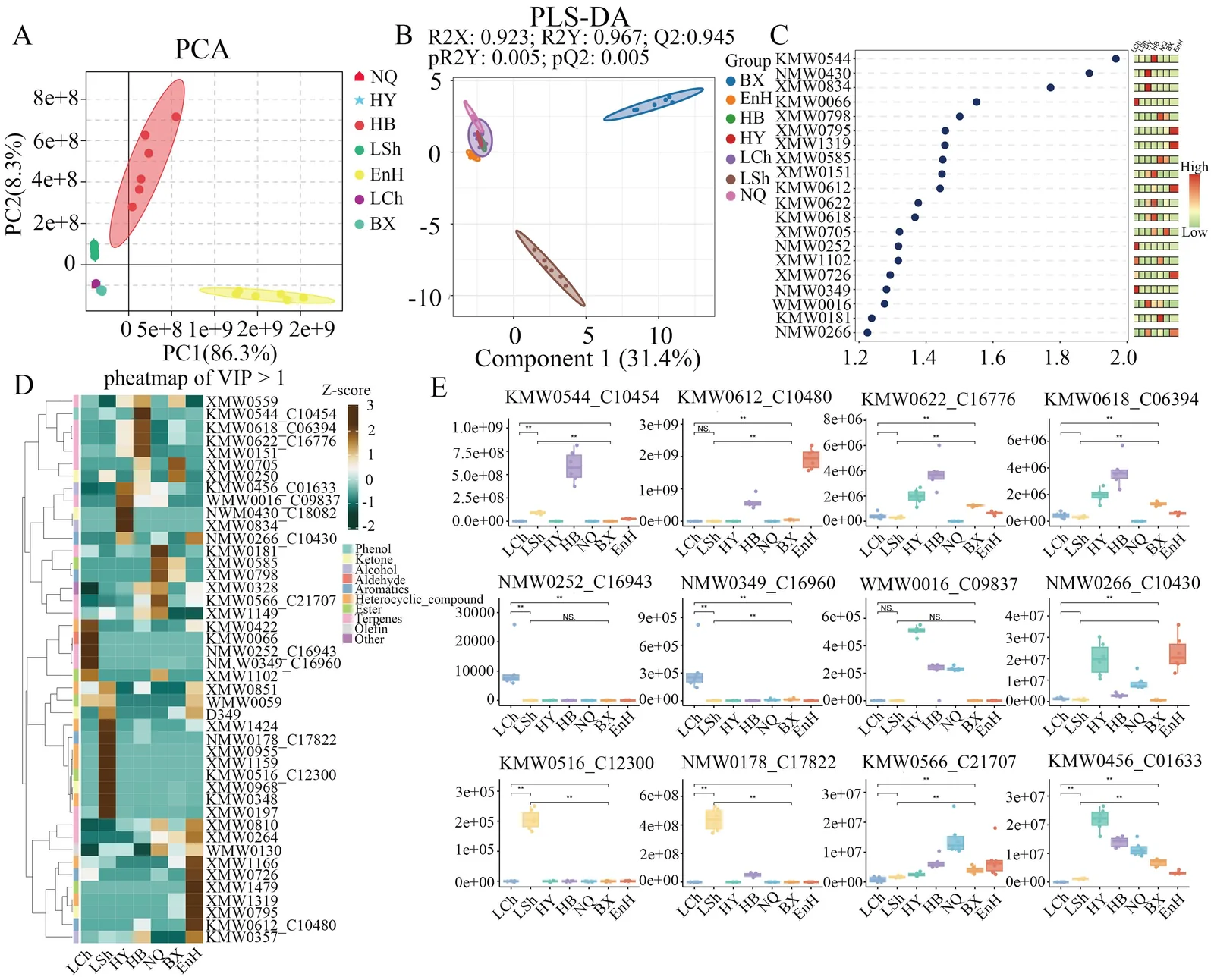
Multi-modal analysis of metabolomic variations in *A. sieboldii* across geographical regions. **(A)** PCA score plot. **(B)** PLS-DA classification model. **(C)** Screening of metabolites (VIP>1). **(D)** Clustering heatmap of key metabolites (VIP > 1). **(E)** Differential abundance of 12 marker metabolites.

To characterize the differential volatile metabolites among *A. sieboldii* originating from distinct geographical origins, we utilized a multivariate analytical approach, which incorporated both principal component analysis (PCA) and partial least squares-discriminant analysis (PLS-DA). The PCA score plot (**Figure 5A**) demonstrated clear separation of samples into four distinct clusters corresponding to the LSh, NQ, EnH, and combined LCh-BX-NQ-HY groups, indicating significant metabolic differences among these populations. Further discrimination was achieved through PLS-DA modeling (**Figure 5B**), which successfully resolved the overlapping LCh-BX-NQ-HY groups using 20 key discriminatory metabolites with variable importance in projection (VIP) scores >1, exhibiting excellent explanatory power and predictive power (R^2^Y 0.967, Q^2^ 0.945) (Wang et al., 2024). Notably, methyl eugenol (KMW0544), 2H-Furo[2,3-h]-1-benzopyran-2-one,5-methoxy-(NMW0430), 3,5-Dihydroxy-4’-methoxydiphenyl (XMW0834) exhibited particularly strong discriminatory power between these groups (**Figure 5C**). Among these, methyl eugenol represents a widely distributed phenylpropanoid across numerous angiosperm families, with especially high abundance in aromatic and therapeutically relevant species. This compound has been extensively documented in the literature for its potent inhibitory effects against fungal pathogens, a property that has been recognized for decades (Tan and Nishida, 2012).

Furthermore, cluster analysis of VIP-selected metabolites (VIP>1) revealed distinct accumulation patterns among the different populations (**Figure 5D**). The LSh group showed significantly higher levels in specific compounds, such as heterocyclic compounds,1H-1,2,3-Triazole (XMW1424), 4-Formyl-3,5-dimethyl-1H-pyrrole-2-carbonitrile (XMW0955), 5H-Oxazolo[3,2-a]pyridine-8-carbonitrile, 6-ethyl-2,3-dihydro-2,7-dimethyl-5-oxo- (XMW1159), Pyrazine, 3,5-diethyl-2-methyl- (KMW0348), while terpenoid metabolites including Naphthalene, 1,2,3,5,6,8a-hexahydro-4,7-dimethyl-1-(1-methylethyl)-, (1S-cis)- (KMW0618), Cyclohexene, 3-(1,5-dimethyl-4-hexenyl)-6-methylene-, [S-(R*,S*)]- (KMW0622), Epizonarene (XMW0151) were markedly enriched in the HB group compared to other populations, among which Epizonarene was reported to be an important ingredient in essential oils with antioxidant and antimicrobial activities (Ibok et al., 2023). In addition, statistical analysis identified several population-specific marker metabolites, identified in all *A. sieboldii* species (**Figure 5E**, **Table 3**).

**Table 3:** Information of the marker metabolites in *A. sieboldii*.

Index	Compounds	Exact mass (Da)	Formula	Class	CAS	RT	RI
KMW0544_C10454	Methyleugenol	178.099	C11H14O2	Phenol	93-15-2	23.03	1412.33
KMW0612_C10480	1,3-Benzodioxole, 4-methoxy-6-(2-propenyl)-	192.079	C11H12O3	Aromatics	607-91-0	24.4	1466.97
KMW0622_C16776	Cyclohexene, 3-(1,5-dimethyl-4-hexenyl)-6-methylene-, [S-(R*,S*)]-	204.188	C15H24	Terpenes	20307-83-9	26.05	1533.94
KMW0618_C06394	Naphthalene, 1,2,3,5,6,8a-hexahydro-4,7-dimethyl-1-(1-methylethyl)-, (1S-cis)-	204.188	C15H24	Terpenes	483-76-1	25.99	1531.56
NMW0252_C16943	(5R,6R)-3,6-Dimethyl-5-(prop-1-en-2-yl)-6-vinyl-6,7-dihydrobenzofuran-4(5H)-one	230.131	C15H18O2	Terpenes	20493-56-5	31.45	1772.29
NMW0349_C16960	Furanodienone	230.131	C15H18O2	Terpenes	24268-41-5	27.88	1611.09
WMW0016_C09837	(-)-Bornyl acetate	196.146	C12H20O2	Terpenes	5655-61-8	19.71	1288.35
NMW0266_C10430	.beta.-Asarone	208.11	C12H16O3	Aromatics	5273-86-9	29.35	1675.95
KMW0516_C12300	.alpha.-Terpinyl acetate	196.146	C12H20O2	Ester	80-26-2	21.32	1347.92
NMW0178_C17822	Benzene, 1,2,3-trimethoxy-5-methyl-	182.094	C10H14O3	Aromatics	6443-69-2	23.13	1416.35
KMW0566_C21707	Naphthalene, decahydro-4a-methyl-1-methylene-7-(1-methylethylidene)-, (4aR-trans)-	204.188	C15H24	Terpenes	515-17-3	24.17	1457.87
KMW0456_C01633	1-Decanol	158.167	C10H22O	Alcohol	112-30-1	25.05	1492.66

The HB group contained significantly elevated levels of Methyleugenol (KMW0544_C10454), Cyclohexene, 3-(1,5-dimethyl-4-hexenyl)-6-methylene-, [S-(R*,S*)]- (KMW0622_C16776), and Naphthalene, 1,2,3,5,6,8a-hexahydro-4,7-dimethyl-1-(1-methylethyl)-, (1S-cis)- (KMW0618_C06394) (*p* < 0.05). Similarly, 1,3-Benzodioxole, 4-methoxy-6-(2-propenyl)- (KMW0612_C10480) was uniquely abundant in the EnH group, while (5R,6R)-3,6-Dimethyl-5-(prop-1-en-2-yl)-6-vinyl-6,7-dihydrobenzofuran-4(5H)-one(NMW0252_C16943) and Furanodienone (NMW0349_C16960) were characteristic of the LCh group. Abdul-Hussain et al., found that the crude aqueous extract of the soil *P. mirabilis* isolate (the highest GC-MS peak is 1,3-Benzodioxole, 4-methoxy-6-(2-propenyl)-) might be a potential producer of QS inhibitors with antibacterial activities (Abdul-Hussain and Lafta, 2024). The LSh population was distinguished by high concentrations of.alpha.-Terpinyl acetate (KMW0516_C12300) and Benzene, 1,2,3-trimethoxy-5-methyl- (NMW0178_C17822). Benny et al., analyzed the phytochemicals present in hexane extract of leaves of *Acrostichum aureum* Linn, and revealed that alpha.-Terpiny acetate was a key ingredient contributing to its medicinal properties (Benny et al., 2026). These results demonstrate that specific volatile metabolites can serve as reliable chemical markers for geographical authentication of *A. sieboldii*.

### 3.5 Model evaluation and variable importance analysis

Prior to habitat-suitability projection, MaxEnt parameter optimization was conducted using ENMeval. Among the 48 candidate combinations of feature classes and regularization multipliers, the model with linear, quadratic, hinge, product, and threshold features (FC = LQHPT) and a regularization multiplier of 3.5 (RM = 3.5) had the lowest AICc value (2654; ΔAICc = 0) and was therefore selected as the optimal model (**Supplementary Figure 7**, **Supplementary Table 3**). Model performance was further evaluated using validation AUC, AUCdiff, and the 10% training omission rate (OR10). AUCdiff represents the difference between training and validation AUC values, with smaller values indicating less discrepancy between model fitting and predictive performance, whereas OR10 represents the proportion of validation occurrences omitted at the 10% training-presence threshold. Compared with the default model (FC = LQHP, RM = 1), the optimized model showed a slightly higher validation AUC (0.844 vs. 0.836), together with lower AUCdiff (0.091 vs. 0.110) and OR10 (0.207 vs. 0.241). Moreover, the AICc value decreased markedly from 2880 to 2654, while ΔAICc decreased from 225 to 0 (**Table 4**). These results indicate that parameter optimization improved model support and predictive performance while reducing the apparent risk of overfitting. Therefore, the optimized LQHPT–3.5 parameter combination was used for all subsequent current and future habitat-suitability projections.

**Table 4:** Comparison of the optimized parameter and default parameter.

Type	FC	RM	OR10	AICc	Delta_AICc	AUCdiff	Test_AUC
Default	LQHP	1	0.241	2880	225	0.110	0.836
Optimized	LQHPT	3.5	0.207	2654	0	0.091	0.844

**Figure 6:**
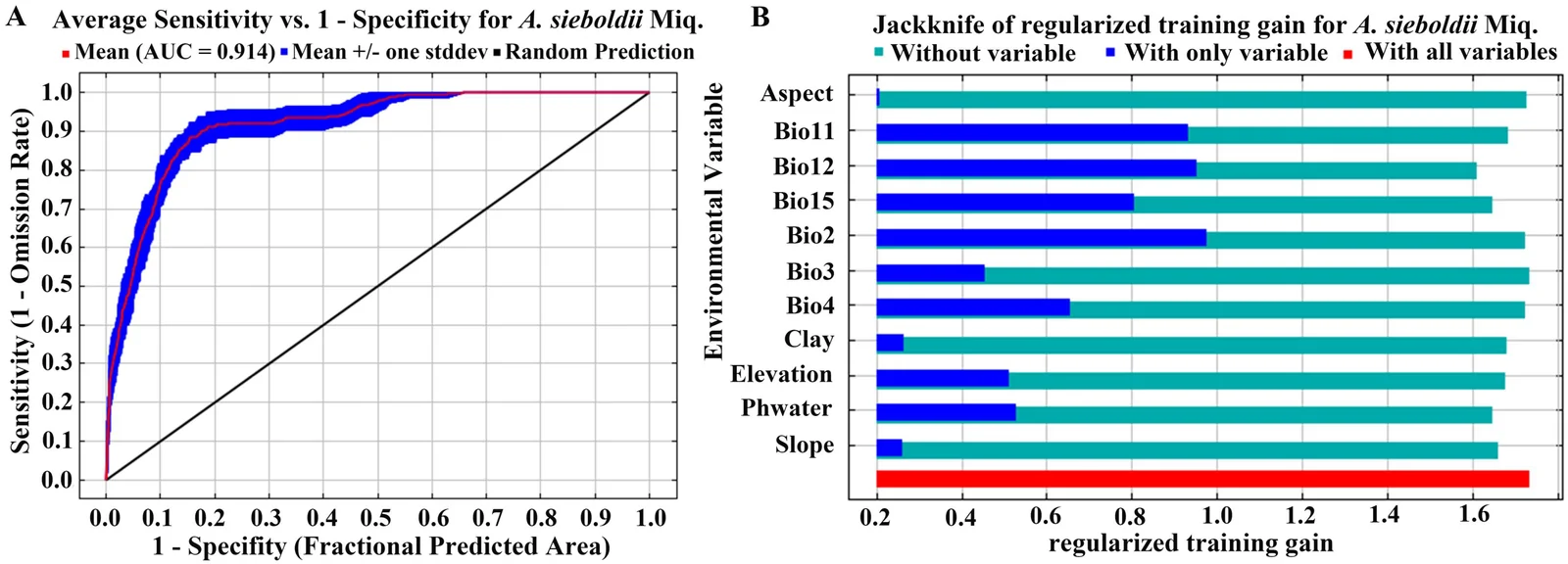
Assessment of MaxEnt model and environmental variables. **(A)** Model validation via the ROC curve and area under the curve (AUC) metric. **(B)** Analysis of variable importance using the jackknife procedure.

The potential distribution of *A. sieboldii* under various future climate scenarios was modeled using MaxEnt, based on 115 spatially filtered occurrence records and 11 selected environmental variables. The model exhibited high predictive accuracy, with an average area under the curve (AUC) value of 0.914 for the receiver operating characteristic (ROC) curve (**Figure 6A**), indicating good discriminatory performance (Zhu et al., 2025a). Variable importance, assessed via a jackknife test of regularized training gain, revealed that climatic factors, particularly Bio2 (mean diurnal range), Bio12 (annual precipitation), and Bio11 (mean temperature of the coldest quarter) exerted stronger influences on the species’ potential distribution than topographic or soil variables (**Figure 6B**), underscoring the predominant role of climate in shaping its suitable habitat. This finding aligns with a growing body of evidence indicating that climate change is a key driver of shifts in species distribution ranges (Montràs-Janer et al., 2024). Specifically, the high sensitivity of many plant species to extreme low temperatures and water availability, mirrored here by the importance of Bio11 and Bio12, has been widely documented; changes in these factors directly constrain survival, recruitment, and dispersal. For example, a recent large-scale study on plant species in northern China highlighted temperature seasonality, annual precipitation, and coldest quarter temperature as primary drivers of habitat dynamics (Sun et al., 2025). Similarly, research on tree ferns in Nepal identified Bio11 and Bio12 as the most significant variables, jointly explaining over 50% of the distribution pattern (Kunwar et al., 2025). The consistency between our results and these independent studies reinforces the reliability of our projections and underscores the potential vulnerability of *A. sieboldii* to future climate shifts, which may lead to range contraction, fragmentation, or migration.

### 3.6 Prediction of suitable areas and response to climate change

The model-predicted current potential distribution of *A. sieboldii* was primarily concentrated in the provinces of Shaanxi, Hubei, Henan, Guizhou, Jiangxi, Zhejiang, Liaoning, and Jilin. Among these, the highly suitable habitats were predominantly identified in Shaanxi, Hubei, and Henan, which aligned with the regions containing the highest density of occurrence records (**Supplementary Figure 1**, **Supplementary Figure 8**). This consistency between model predictions and empirical data supports the robustness of the modeling approach. Spatially, the suitable habitats exhibited a concentric pattern, with habitat suitability gradually decreasing from the highly suitable core areas to the marginally suitable peripheries (**Supplementary Figure 8**). This pattern likely reflects the spatial gradient of the key climatic determinants identified, such as temperature and precipitation, with optimal conditions concentrated in the core region becoming less favorable toward the edges.

**Figure 7:**
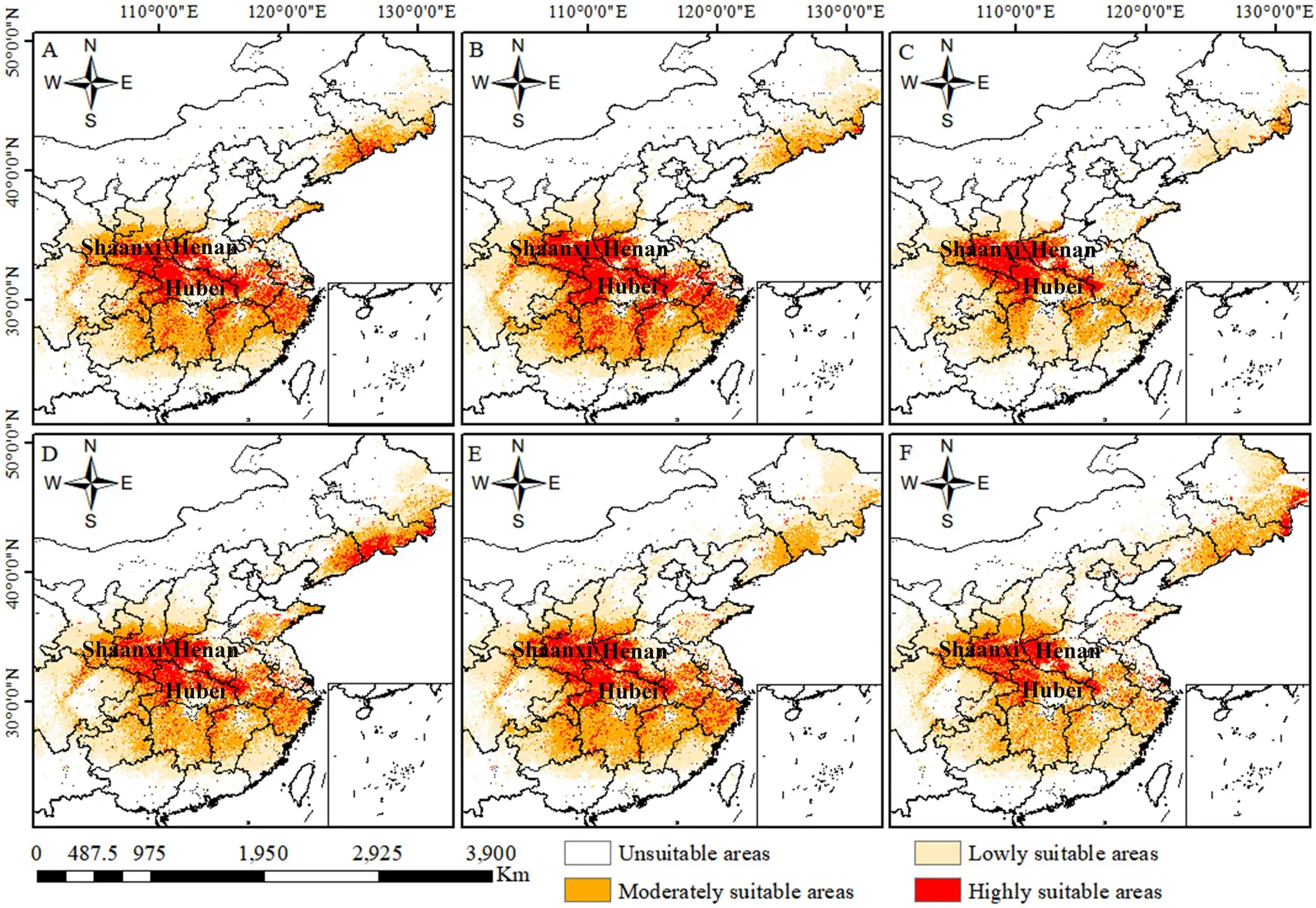
Projected potential distribution of *A. sieboldii*. under future climate scenarios. **(A–C)** Distribution under the SSP1-2.6 scenario for the 2050s **(A)**, 2070s **(B)**, and 2090s **(C)**. **(D–F)** Distribution under the SSP5-8.5 scenario for the 2050s **(D)**, 2070s **(E)**, and 2090s **(F)**.

Future potential distributions of *A. sieboldii* for the 2050s, 2070s, and 2090s were projected under the SSP1-2.6 and SSP5-8.5 climate scenarios. The projections showed that highly suitable habitats remained consistently concentrated in Shaanxi, Hubei, and Henan provinces across different future scenarios and time periods (**Figure 7**), indicating relatively high habitat stability in these regions and suggesting a comparatively close match between future environmental conditions and the ecological requirements of *A. sieboldii* (Liu et al., 2024a). Climate change, by increasing mean temperatures and the frequency of extreme events, is causing habitat loss and degradation, thereby exacerbating the biodiversity crisis and shifting the suitable habitats for species (Pörtner et al., 2023). Numerous studies have demonstrated that climate change profoundly affects plant growth, physiology, and distribution (Ostad-Ali-Askari et al., 2020). For example, Zhao et al. projected that the suitable habitat area for *Pinus massonianawould* decreased by approximately 44% compared to baseline climatic conditions, accompanied by severe habitat fragmentation (Zhao et al., 2024). Similarly, Chang et al. reported that for the endangered conifer *Taxus cuspidata*, an increase in suitable habitat is only projected under the SSP126 scenario by the 2070s (Chang et al., 2024).

In this study, the overall extent of unsuitable areas exhibited limited variation under climate change, the area of highly suitable habitat and moderately suitable habitat showed a marked declining trend over time (**Supplementary Table 11**). This reduction was particularly pronounced under the high-emission SSP5-8.5 scenario, where the highly suitable area decreased from approximately 292,136.5 km² in the 2050s to 272,959.6 km² in the 2070s, and further to 237,149.7 km² in the 2090s, and the moderately suitable area decreased from 742,740.5 km^2^ in 2050s to 633,656.3 km^2^, and further to 645,753.0 km^2^ in 2090s (**Supplementary Table 11**), indicating a significant negative impact of rising temperatures on the overall suitable habitat area for the species.

The projected habitat areas showed distinct temporal responses under the two future emission scenarios (**Supplementary Table 11**). Under SSP1-2.6, the highly suitable area decreased from 577,032.8 km² under current conditions to 431,089.1 km² in the 2050s, temporarily increased to 535,672.6 km² in the 2070s, and then declined markedly to 295,552.1 km² by the 2090s. The moderately suitable area initially increased to 855,863.0 km² in the 2050s but subsequently decreased to 806,550.9 km² in the 2070s and 521,192.1 km² in the 2090s. Under SSP5-8.5, the moderately suitable area remained higher than that under current conditions, reaching 864,988.9, 913,287.0, and 871,784.4 km² in the 2050s, 2070s, and 2090s, respectively, whereas the highly suitable area declined to 404,316.1 km² in the 2050s, remained relatively stable at 407,073.5 km² in the 2070s, and decreased further to 294,751.6 km² in the 2090s. Overall, the projections suggest a redistribution of habitat-suitability classes under future climate scenarios, with a general reduction in highly suitable habitat and the most pronounced losses occurring by the 2090s.

**Figure 8:**
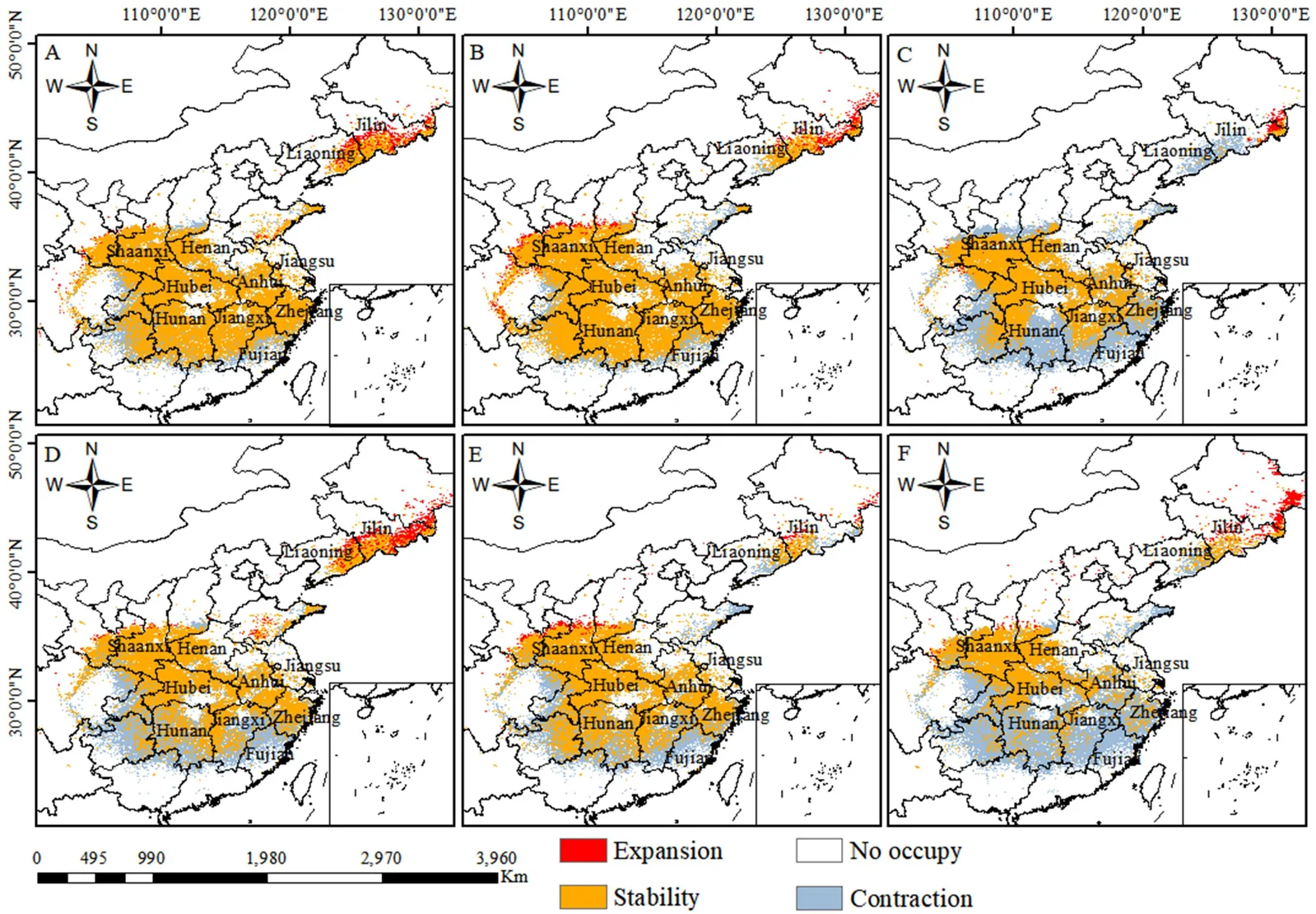
Spatial-temporal changes in the potential distribution of *A. sieboldii* Miq. Projections under SSP1-2.6 and SSP5-8.5 for the 2050s, 2070s, and 2090s, relative to the current distribution. **(A–C)** Changes under SSP1-2.6 for the 2050s **(A)**, 2070s **(B)**, and 2090s **(C)**. **(D–F)** Changes under SSP5-8.5 for the 2050s **(D)**, 2070s **(E)**, and 2090s **(F)**.

To assess the distributional shifts of *A. sieboldii* under future climate scenarios, the projected future distributions were compared with the current baseline and classified into expansion, contraction, and stable areas (**Figure 8**, **Supplementary Table 12**). Under the SSP1-2.6 scenario, stable suitable areas in the 2050s and 2070s were mainly distributed in Shaanxi, Hubei, Henan, Hunan, Jiangxi, Anhui, and Zhejiang provinces (**Figure 8**). Notably, in Shaanxi Province, the relatively stable habitats were concentrated mainly in the central and southern parts of the province, spatially corresponding to the Qinling Mountain region. The persistence of suitable habitat in this area may be associated with the pronounced topographic and environmental heterogeneity of the Qinling Mountains, which can provide diverse local climatic and ecological conditions favorable for plant persistence (Zhu et al., 2025b). This interpretation is also consistent with previous studies highlighting the Qinling Mountains as an important center of plant diversity and endemism in China, supporting more than 1,620 endemic plant species (Xu et al., 2019). By the 2090s, however, the extent of stable habitat was substantially reduced, accompanied by pronounced contraction in parts of the southern distribution, particularly in Jiangxi and Hunan provinces. A broadly similar pattern was observed under SSP5-8.5, although the magnitude of spatial change differed among time periods. Under this scenario, stable habitat remained extensive in the 2050s and 2070s but declined considerably by the 2090s, while habitat contraction became more pronounced (**Figure 8**).

Range expansion was projected mainly toward the northern part of the current suitable distribution, including parts of Liaoning and Jilin provinces, whereas contraction occurred predominantly in the southern portion of the suitable range (**Figure 8**). These changes highlight a general trend, predominant expansion areas in the northern suitable habitats, contraction areas in the southern suitable habitats (Zhu et al., 2025a), and this pattern was found in previous studies on other species, including Chinese *Ziziphus jujuba* Mill (Zhao et al., 2021), *Stipa purpurea* Griseb (Ma et al., 2022b), which were tend to migrate to the north to avoid the negative effect of increasing temperature and the south of the suitable habitats usually suffered huge contractions and habitats loss. Nevertheless, the magnitude of expansion varied among scenarios and time periods and remained consistently smaller than that of contraction. Under both SSP1-2.6 and SSP5-8.5, contraction exceeded expansion throughout the future periods examined, with the imbalance becoming particularly pronounced by the 2090s (**Supplementary Table 12**). Overall, these results indicate a net reduction and spatial redistribution of suitable habitat for *A. sieboldii* under future climate scenarios, characterized primarily by contraction in southern areas and more limited expansion toward northern regions.

We further analyzed shifts in the centroid of the suitable habitat of *A. sieboldii* under different future climate scenarios. Compared with the current centroid located in Hubei Province, the projected centroids generally shifted northward toward the Hubei–Henan region and Hubei Province, although the direction and magnitude of displacement varied among scenarios and time periods (**Supplementary Figure 9**). Under SSP1-2.6, centroid displacement was relatively limited, with the projected centroids remaining comparatively close to the current distribution center throughout the three future periods. In contrast, greater spatial variation was observed under SSP5-8.5. The centroid shifted northeastward in the 2050s, showed a comparatively smaller displacement in the 2070s, and moved markedly northward into Henan Province by the 2090s (**Supplementary Figure 9**). Overall, the centroid trajectories indicate a general tendency toward northward redistribution of suitable habitat under future climate change, with a more pronounced displacement under the higher-emission SSP5-8.5 scenario, particularly by the 2090s. This pattern is consistent with recent evidence that climate warming can drive the redistribution of terrestrial plant species toward cooler environments, including higher latitudes and elevations (Chan et al., 2024).

### 3.7 Conservation strategies

Wild Chinese medicinal plants are valued for their significant pharmaceutical properties, yet they face growing threats from anthropogenic pressures, such as overharvesting, and habitat degradation exacerbated by recent climate warming. These changes not only reduce suitable habitats but may also alter the accumulation of bioactive compounds essential to their medicinal efficacy. For example, *Sophora alopecuroides* L., a perennial herb in northwest China, plays dual ecological roles in windbreaking and sand fixation while possessing considerable medicinal value. However, shifts in its habitat due to climate and human activities risk affecting its natural product profiles (Rong et al., 2024). Similarly, studies on ironwort (*Sideritis*), an important medicinal resource in Mediterranean regions, show that rising temperatures have reduced habitat suitability by approximately 50% across major mountain ranges (Theodoridis et al., 2024). These cases underscore how climate-driven habitat loss compromises the survival of medicinal plants globally. With rising temperatures, the suitable habitat of *Asarum sieboldii* is projected to contract over time. To conserve this valuable wild medicinal plant, the following strategies are proposed. First, based on the projected habitat changes, Shaanxi, Hubei, and Henan provinces retained relatively stable suitable habitats across future climate scenarios and should therefore be prioritized for *in situ* conservation, habitat protection, and long-term population monitoring. Second, newly suitable areas projected in northern regions, such as parts of Liaoning Province, may provide potential opportunities for future cultivation; however, field validation and small-scale introduction trials should be conducted before establishing large-scale cultivation bases. Third, in regions projected to experience substantial habitat contraction, particularly Jiangxi and Hunan provinces, *ex situ* conservation measures, including germplasm collection and seed banking, are recommended to reduce the risk of genetic-resource loss.

## 4 Conclusion

This study comprehensively characterized volatile-metabolite variation in *Asarum* species, with particular emphasis on *A. sieboldii*, using HS-SPME/GC–MS combined with multivariate statistical analyses. A total of 131 volatile metabolites belonging to 11 chemical classes were annotated, with terpenoids representing the dominant group (47.3%). Comparative analyses revealed pronounced differences in volatile profiles among the sampled *Asarum* taxa, and five candidate discriminatory metabolites showed potential for distinguishing *A. sieboldii* from the five related species examined. Geographical analysis further revealed substantial variation among the seven sampled populations of *A. sieboldii*, with 20 metabolites (VIP > 1) contributing markedly to population differentiation and several metabolites showing region-associated patterns. These findings indicate that volatile-metabolite profiling has considerable potential for species authentication and geographical origin traceability of *A. sieboldii* medicinal materials. In parallel, MaxEnt modeling was used to assess the current and future habitat suitability of *A. sieboldii*. Climatic variables showed greater importance in predicting habitat suitability than topographic and soil variables. Under the future climate scenarios examined for the 2050s, 2070s, and 2090s, relatively stable suitable habitats were consistently projected in Shaanxi, Hubei, and Henan provinces. Overall, suitable habitat for *A. sieboldii* was projected to decrease, with habitat contraction exceeding expansion. These projections provide ecological information for identifying relatively stable conservation areas, assessing regions potentially vulnerable to habitat loss, and informing future cultivation planning under climate change.

A limitation of this study is that each of the five congeneric species was represented by only one population, whereas *A. sieboldii* was sampled from multiple populations. Consequently, the observed interspecific differences in volatile profiles may partly reflect population-specific or local environmental effects, and the identified metabolites should therefore be regarded as candidate markers for distinguishing the sampled taxa rather than as universally validated species-specific biomarkers. Overall, this study provides complementary chemical and ecological information for *Asarum* medicinal resources. Volatile-metabolite profiling provides candidate markers for species authentication and geographical origin traceability, while habitat-suitability modeling provides ecological evidence to support climate-informed conservation and cultivation planning of *A. sieboldii*.

## Data Availability

The raw metabolomics data generated in this study have been deposited in the OMIX repository of the National Genomics Data Center (NGDC, https://ngdc.cncb.ac.cn/) under accession number OMIX019483. The original contributions presented in the study are included in the article/**Supplementary Material**.
