# Supplementary material for: Comparative volatile profiling of Asarum species and MaxEnt-based habitat assessment of Asarum sieboldii: implications for species authentication, geographical origin traceability, and climate-informed conservation
Source: Front Plant Sci. 2026 Sep 1;17:1937801. doi: 10.3389/fpls.2026.1937801 (PMC13574818; doi:10.3389/fpls.2026.1937801)
Supplement: Supplementary file 1 [file DataSheet1.docx]

**Figure S1.** Geographic distribution of *Asarum sieboldii* Miq.

**Figure S2.** Heatmap visualizing the pairwise correlations among environmental variables.Correlations are calculated based on Pearson correlation coefficients, with red and blue colors representing positive and negative correlations, respectively.

**Figure S3.** Variable importance in projection (VIP) scores of 30 discriminatory volatile metabolites among six *Asarum* species identified by PLS-DA.

**Figure S4.** Volcano plot analysis between *A. heophilum*, *A. himalaicum*, *A. macranthum*, *A. renicordatum*, *A. splendens*, and *A. sieboldii.*

**Figure S5.** Permutation tests of pairwise OPLS-DA models between *A. sieboldii* and five related *Asarum* species. The OPLS-DA models were validated using 200 permutation tests. (A) *A. geophilum* vs *A. sieboldii*; (B) *A. himalaicum* vs *A. sieboldii*; (C) *A. macranthum* vs *A. sieboldii*; (D) *A. renicordatum* vs *A. sieboldii*; and (E) *A. splendens* vs *A. sieboldii*. The distributions of R²Y and Q² values obtained from randomly permuted class labels are shown, together with the corresponding values of the original models.

**Figure S6.** Differential analysis between *A. heophilum*, *A. himalaicum*, *A. macranthum*, *A. renicordatum*, *A. splendens*, and *A. sieboldii* visualized by volcano plot.

**Figure S7.** Variation in ΔAICc among 48 candidate MaxEnt models with different feature-class (FC) and regularization-multiplier (RM) combinations. The optimal model was obtained with FC = LQHPT and RM = 3.5, corresponding to the minimum AICc (ΔAICc = 0).

**Figure S8.** Prediction of current potential distribution area of *Asarum sieboldii.*

**Figure S9.** Migratation of centroid of suitable habitats.
